# GH Alters Lymphatic Vessels in Female Mice and STAT5 Phosphorylation in Human Lymphatic Endothelial Cells

**DOI:** 10.1210/endocr/bqaf194

**Published:** 2026-01-19

**Authors:** Christopher Walsh, Emily Scott, Elise Wagner, Jerome Walsh, Shashank Reddy, Arshad Ahmad, Reetobrata Basu, Eva Sevick-Muraca, Rich Brody, Uday Sandbhor, Sebastian Neggers, John J Kopchick

**Affiliations:** Heritage College of Osteopathic Medicine, Ohio University, Athens, OH 45701, USA; Institute for Molecular Medicine and Aging, Ohio University, Athens, OH 45701, USA; Department of Biological Sciences, Ohio University, Athens, OH 45701, USA; Department of Biological Sciences, Ohio University, Athens, OH 45701, USA; Heritage College of Osteopathic Medicine, Ohio University, Athens, OH 45701, USA; Heritage College of Osteopathic Medicine, Ohio University, Athens, OH 45701, USA; Institute for Molecular Medicine and Aging, Ohio University, Athens, OH 45701, USA; Heritage College of Osteopathic Medicine, Ohio University, Athens, OH 45701, USA; Institute for Molecular Medicine and Aging, Ohio University, Athens, OH 45701, USA; The Brown Foundation Institute of Molecular Medicine, The University of Texas Health Science Center, Houston, TX 77030, USA; Early Drug Development, Infinix Bio LLC, Columbus, OH 43212, USA; Early Drug Development, Infinix Bio LLC, Columbus, OH 43212, USA; Department of Medicine, Endocrinology, Erasmus Medical Center, 3015 GD, Rotterdam, The Netherlands; Heritage College of Osteopathic Medicine, Ohio University, Athens, OH 45701, USA; Institute for Molecular Medicine and Aging, Ohio University, Athens, OH 45701, USA

**Keywords:** growth hormone, lymphatic system, lymphangiogenesis, transgenic, lymphatic pumping

## Abstract

Disruption of lymphatic function underlies a broad spectrum of inflammatory and metabolic disorders, yet the hormonal pathways that regulate lymphatic biology remain poorly defined. GH, which is implicated in similar disease states, has an unclear role in lymphatic homeostasis. To address this gap, we investigated how chronic alterations in GH signaling alter lymphatic structure and function. Using transgenic mouse lines with increased, decreased, or absent GH action, we quantified the effect of GH on lymphatic pumping rate and lymphangiogenic remodeling during wound healing using near-infrared fluorescent imaging. We also measured markers of lymphatic endothelial cells using Western blot and immunohistochemistry across multiple mouse organs. Lymphatic pumping rate positively correlated with GH action, whereas both elevated and absent GH signaling delayed wound healing. In contrast, the lymphatic vascular density and the expression of protein markers of lymphatic endothelial cells were inversely correlated with GH activity. Additionally, we showed that primary human dermal lymphatic endothelial cells express the GH receptor and exhibit acute GH-activated signaling and that this activation can be blocked with new and Food and Drug Administration-approved GH receptor antagonists. Together, these findings identify GH as a regulator of the lymphatic system and suggest that GH receptor antagonism could be a potential strategy to address lymphatic dysfunction.

The lymphatic vasculature dynamically responds to maintain tissue fluid balance, yet its hormonal regulation remains underexplored relative to that of the blood vasculature (1). GH is essential for blood vessel homeostasis, and perturbations in GH signaling lead to endothelial cell dysfunction, as observed in patients with acromegaly and GH deficiency, who have opposing GH phenotypes but both develop cardiovascular disease with capillary rarefication (2-4). In the acute setting, GH increases blood flow by modulating vascular tone (5) and angiogenesis (6) and increases extracellular hydration through its effects on the kidney (7) and the extracellular matrix (8). The GH receptor (GHR) is abundantly expressed on human lymphatic endothelial cells (LECs) (9) and acutely stimulates lymph flow (10) and lymphangiogenesis (11) in rodent models when activated. Despite these observations, the chronic effects of GH imbalance on lymphatic vessel structure and function remain unexplored, as well as the ability to target GHR activation in human LECs.

The lymphatic system plays a crucial role in resolving inflammation by draining cytokines and cells from the inflammatory microenvironment. Cytokines produced during chronic inflammation disrupt LEC function and diminish the lymphatic network's ability to maintain homeostatic lymphatic drainage (12). Once established, lymphatic dysfunction contributes to a positive feedback loop in which impaired lymph drainage drives cytokine accumulation and tissue remodeling, thereby further compromising lymphatic function and perpetuating chronic inflammation (13, 14). Dysfunction in the lymphatic system is not only an outcome but actively contributes to many common conditions like obesity, cancer, cardiovascular disease, inflammatory bowel disease, and neurodegenerative diseases (15). Although dysfunction in paracrine and autocrine GH signaling has also emerged as a covert player in the previously mentioned diseases (2, 16, 17), the interplay between disrupted GH signaling and lymphatic dysfunction has not been studied.

We hypothesized that chronic GH perturbation drives genotype-specific changes in the lymphatic pumping rate and in the expression of LEC markers and that GHR antagonism influences primary human LEC physiology. We approached these goals by employing transgenic mice with increased, decreased, and no GH action during our assessment. We quantified lymphatic pumping rate and lymphangiogenic remodeling during wound healing, dermal lymphatic vascular density, the expression of LEC marker proteins in multiple organs, and GHR-dependent signaling effects in human primary dermal LECs using 2 GHR antagonists.

## Materials and Methods

### Mice

All animal procedures were carried out in accordance with the Animal Welfare Act set forth by the National Institute of Health and approved by the institutional animal care and use committees at Ohio University and The University of Texas Health Science Center. The various mouse lines were created and propagated on a C57BL/6J background. A bovine GH (bGH) mini-gene was used to generate bGH transgenic mice. A single codon change in the bGH gene, resulting in a G119A amino acid substitution, was used to create GHR antagonist (GHA) transgenic mice (18). The GH receptor knockout mice (GHRKO) were created by introducing a loss-of-function mutation into the gene encoding the GHR (19). Female mice from 3 transgenic lines and their wild-type littermates (n = 6) were bred and maintained at Ohio University in Athens, Ohio, until they were 6 months old and then sent to The University of Texas Health Science Center in Houston, Texas, for near-infrared fluorescent (NIRF) imaging. The mice were dissected when they were 8 months old, 6 weeks after the wounding experiment began.

### NIRF

To prepare for NIRF imaging, mice were shaved and depilated with a depilatory cream (Nair) the day before imaging. Indocyanine Green (ICG) (Diagnostic Green, LLC, Farmington Hills, MI, USA, NDC 70100-424-01) was prepared fresh before each imaging session by dissolving 0.15 mg of dry ICG in 30 μL of sterile water, to improve solubility and then adding 270 μL of 0.9% sodium chloride solution (saline), creating a 662 μM stock solution. This was further diluted with saline to reach a final concentration of 165.5 µM. Mice were anesthetized and maintained on 2% to 3% of vaporized isoflurane in flowing oxygen delivered through a nose cone to the prewarmed imaging stage. Six uL of ICG was intradermally injected into skin lateral to the base of the tail using a 10 uL glass syringe (Hamilton 700 series) and a 32-gauge needle (Hamilton Cat# 7803-14). The creation of an intradermal induration signified successful injection into the dermal space. The injection site was then covered with black adhesive tape to prevent camera saturation during imaging. The custom-built imaging station was outfitted with a laser diode (85 mA and 80 mW, DL7140-201, Sanyo) that emitted 785-nm light, uniformly diffused over a circular area 8 cm in diameter, where the mouse was placed. Fluorescent signals were collected using an electron-multiplying charge-coupled device camera (model 7827-0001, Princeton Instruments) with an 830 nm emission detector and filters that rejected backscattered and reflected emission light. During the imaging sessions, light from the laser diode excites the ICG dye, causing it to emit light at 830 nm, which is then captured by the electron-multiplying charge-coupled device camera. Software written in V++ was used to create and collect image stacks at a rate of 5 frames per second. Stacks of images were loaded into Fiji software (ImageJ distribution, RRID: SCR_002285) for further analysis.

**Figure 1.**
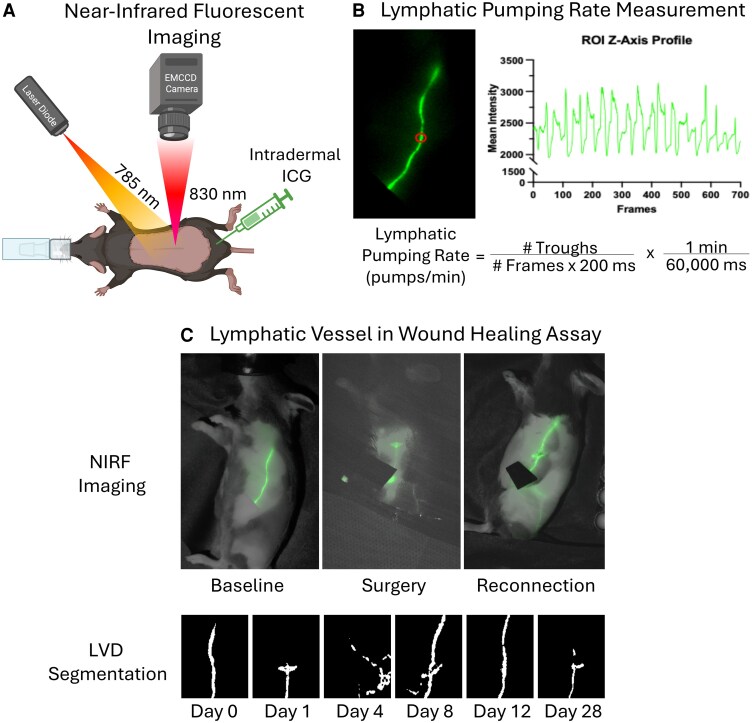
Experimental design using NIRF. (A) Schematic of NIRF imaging using indocyanine green created using Biorender: https://app.biorender.com/illustrations/68756e1aadfb1bc8a4626d04. (B) The lymphatic pumping rate is calculated by plotting the mean intensity of a region of interest (circle) in each frame of a NIRF video, counting the number of troughs, and applying the formula. In this example, the lymphatic pumping rate is 8.1 pumps per minute. (C) NIRF imaging was used to identify and transect the collecting lymphatic vessel of the mouse. Repeated imaging over the preceding month was performed, and image segmentation was used to calculate lymphatic vascular density. Abbreviations: NIRF, near-infrared fluorescent.

To measure the lymphatic pumping rate, a circular region of interest (ROI) was drawn over the conducting lymphatic vessel pumping ICG-laden lymph between the inguinal and axillary lymph nodes in a stack of images. The mean intensity of the ROI in each frame was plotted over time to create a line graph. The number of troughs and frames from each imaging session was counted and inserted into the formula in Fig. 1B to calculate the lymphatic pumping rate. To calculate the baseline pumping rate in this study, the left and right collecting vessels were imaged for at least 3 minutes on different days and then averaged.

### Full-dermis Thickness Wound Healing Model

The wound healing model was performed as previously described (20). Before surgery, each mouse was injected subcutaneously with 0.25% bupivacaine (5 mg/mL) to provide analgesia. NIRF imaging was performed as previously described to visualize the lateral conducting lymphatic vessel. Once the vessel was visualized, sterile surgical attire was donned. The site was sterilized by wiping it 3 times with a new Povidone-Iodine wipe and a 70% ethanol wipe. Sterile surgical scissors were used to open the skin and expose the conducting vessel at the midpoint between the axillary and inguinal lymph nodes. Scissors were used to transect the vessel, and the absence of forward-flowing lymph was confirmed with NIRFLI imaging. The wound was then closed at the skin surface using surgical glue (3 M Vetbond tissue adhesive Cat# 1469SB). NIRF imaging was then performed repeatedly over the next month to assess the healing of the surgically wounded vessel and the impact of the surgery on the unaffected, contralateral lymphatic conducting vessel. Lymphatic vascular density (LVD) was quantified using an averaged projection of the stack of images acquired during the NIRF imaging sessions. The projection was then enhanced using the *Tubeness* plugin (21) and segmented using the Trainable Weka Segmentation plugin (22). The segmented area was divided by the ROI to generate the LVD measure. Due to differences in animal size, a unique ROI was used for each genotype, defined as the average of the ear-to-tail and back-to-belly lengths.

### Immunohistochemistry

At dissection, the hearts and both flanks of skin from 3 mice were dissected, cut along the transverse plane, placed into plastic cassettes, and submerged in 10% neutral-buffered formalin for 24 hours at room temperature. Then the formalin was removed, and the samples were stored in 70% ethanol until they were embedded in paraffin wax 3 months later. Three sections were taken from each flank: 1 from the middle of the flank where the surgical incision occurred, 1 from a centimeter cranial, and 1 from a centimeter caudal to the incision. The wax sections on glass slides were stored at −80 °C. The slides were deparaffinized by washing 3× in xylene for 5 minutes, 1× in 1:1 xylene and water for 3 minutes, 2× in 100% ethanol for 3 minutes, 1× in 95% ethanol for 3 minutes, 1× in 70% ethanol for 3 minutes, 1× in 50% ethanol for 3 minutes, and finally in Tris-buffered saline with 0.05% Tween 20 (TBS-T). Heat-mediated antigen retrieval was performed by placing the slides in 60 mL of universal HIER (Abcam Cat# ab208572) in a Coplin jar, then pressure-cooked on high for 20 minutes in an Instant Pot. Once depressurized, the jars were slowly cooled for 30 minutes using running room-temperature water. The slides were washed twice with TBS-T, the sections were circled with a PAP pen, and the sections were permeabilized using 2 washes of 0.3% TBS-Tween 20 with 0.025% TBS-Triton X-100 for 10 minutes. Then the sections were blocked using TBS-T with 10% normal donkey serum (Abcam Cat# ab7475) and 5% bovine serum albumin (BSA) for 1 hour at room temperature in a humidified incubation chamber. Then the slides were washed twice with TBS-T and incubated in 3% hydrogen peroxide for 15 minutes at room temperature. The sections were rewashed in TBS-T and then incubated with the primary antibodies overnight. The primary antibodies were prepared in TBS-T with 1% BSA at the following concentrations: 4 ug/mL of goat anti-mouse lymphatic vessel-hyaluronic acid receptor 1 (LYVE1; R&D Systems Cat# AF2125, RRID: AB_2297188), 3 ug/mL of rabbit anti-mouse CD31 (Proteintech Cat# 28083-1-AP, RRID: AB_2881055), or 4 ug/mL goat isotype control antibody (R&D Systems Cat# AB-108-C, RRID: AB_354267) or 3 ug/mL rabbit isotype control antibodies (R&D Systems Cat# AB-105-C, RRID: AB_354266). The sections were washed 3 times for 5 minutes in TBS-T and incubated with the secondary antibodies for 1 hour at room temperature. The secondary antibodies were mixed in 1% BSA in TBS-T at 1:200 for the red donkey anti-goat IgG Cy5 (Abcam Cat# ab6566, RRID: AB_955056) and 1:400 for the green donkey anti-rabbit IgG Alex Fluor 400 (Abcam Cat# ab150073, RRID: AB_2636877). The sections were washed twice more in TBS-T, and then the skin sections were incubated in 4',6-diamidino-2-phenylindole (DAPI), diluted 1:1000 in TBS-T (FluroPure, ThermoFisher Cat# D211490). The sections were mounted using Vectashield Vibrance Antifade Mounting Media (Vector Laboratories Cat# H-1700-2). Imaging was performed using a fluorescent microscope (Zeiss Axio Observer, RRID: SCR_021351) with an AxioCam 305 color camera, operated with Zen 2.6 software (RRID: SCR_021725). Each section was imaged 3 to 5 times with a 20× objective with 0.8 aperture. Images were processed using QuPath (RRID: SCR_018257) image analysis software. Quantitative measures of LYVE1 were obtained by training a pixel classifier in QuPath to distinguish LYVE1 staining from background and nonspecific staining. The same classifier was then used to quantify the LYVE1-staining area and the image's total area.

### Cell Culture

Primary human dermal LECs were purchased from a distributor who isolated them from human foreskin and characterized them as over 95% positive by immunofluorescence for cytoplasmic factor VIII, cytoplasmic uptake of Di-I-Ac-LDL, and cytoplasmic PECAM1 (Angio-Proteomie Cat# cAP-0003). Cells were cultured according to the distributor's instructions in a humidified incubator at 37 °C and 5% CO_2_ with endothelial growth media (Angio-Proteomie Cat# cAP-02) (EGM), which consisted of endothelial basal media with 10% fetal bovine calf serum, 10% endothelial growth factors, and 1% penicillin-streptomycin. Cells were passed every 7 days to a new T75 flask coated with Matrigel coating solution (Angio-Proteomie Cat# cAP-58). Cells were used up until the tenth passage. The cells were treated with recombinant human GH (Antibodies-online Cat# ABIN2017921); pegvisomant, a Food and Drug Administration-approved GHR antagonist used in the treatment of patients with acromegaly (23); and compound D, a novel 191-amino-acid GHR antagonist with 2 site-directed pegylation sites (24). The combination of treatments consisted of the following: EGM, EGM without the 10% FBS (REGM), endothelial basal media with 1% penicillin-streptomycin (EBM), REGM with pegvisomant at 500 nM (10 ug/mL) (Peg), REGM with compound D at 500 nM (10 ug/mL) (CpD), REGM with human GH 5 nM or 100 ng/mL (GH), REGM with human GH at 5 nM and pegvisomant at 500 nM (GH + Peg), and REGM with human GH at 5 nM and compound D at 500 nM (GH + CpD).

To measure GH activation, cells were plated in 6-well plates and, once at 80% confluence, were starved for 12 hours and then treated with the aforementioned treatments for 10 minutes. Immediately after the plates were washed with cold PBS and stored at −80 °C until the proteins were extracted. For the migration and invasion assays, the transwell CytoSelect 24-Well Cell Migration and Invasion Assay Combo Kit with 8-micrometer pores (CellBioLabs Cat# CBA-100-C) was used, and the manufacturer's instructions were followed. The cells were pretreated with the aforementioned treatments for 48 hours and plated at 210 000 cells per well in EBM. EGM was placed into the bottom well, and cells were allowed to migrate for 18 hours and invade for 36 hours before the experiment was stopped and the measurements were made. Multiple images were taken of the cells on the bottom before colorimetric quantification. Instead of pretreatment, cells were mixed with the treatment media at the beginning of the tube formation assay, which was performed using the 15-well u-slide chambered coverslips (Ibidi Cat# 81506) with growth factor-reduced Matrigel (Corning Cat# 256231). Cells were grown until 80% confluency in EGM, split, resuspended in the treatment media with 2% BSA, and plated at 7000 cells per well. After 17 hours, the wells were imaged in phase contrast on a Zeiss Axio Observer microscope using an AxioCam 305 camera and a 20× objective. Images were then stitched together using Zen 2.6 software. Stitched images were quantified using the ImageJ plugin *Angiogenesis Analyzer* (25).

### Western Blot Analysis

Proteins were extracted from tissue samples using a 2× radioimmunoprecipitation assay buffer buffer with 1:100 protease inhibitor and 1:100 phenylmethylsulfonyl fluoride in a Precellys homogenizer (RRID: SCR_026287) assisted with ceramic beads. For cultured cells, the same extraction buffer was used, but a cell scraper was used to lyse and dissociate the cells. Samples were purified by centrifugation and sonication, and protein concentrations were measured with the Bradford assay. The LEC-specific proteins used were against prospero homeodomain protein 1 (1:1000, Abcam Cat# ab199359, RRID: AB_2868427), LYVE1 (1:1000, R&D Systems Cat# AF2125, RRID: AB_2297188), vascular endothelial growth factor receptor 3 (1:1000, Thermo Fisher Scientific Cat# 14-5988-82, RRID: AB_467795), and podoplanin (1:1000, Abcam Cat# ab256559, RRID: AB_2936436).

The antibodies used to assess LEC signaling were against phosphorylated signal transducer and activator of transcription 5 (1:1000, R&D Cat# MAB 41901, RRID: AB_3658258), total signal transducer and activator transcription 5 (STAT5; 1:1000, CST Cat# 25656S, RRID: AB_2798908), phosphorylated signal transducer and activator transcription 3 (1:1000, CST Cat# 9145S, RRID: AB_2491009), total signal transducer and activator transcription 3 (STAT3; 1:1000, CST Cat#12640S, RRID: AB_2629499), phosphorylated p44/42 mitogen-activated protein kinase (1:1000, CST Cat# 9101S, RRID: AB_331646), total p44/42 mitogen-activated protein kinase (1:1000, CST Cat# 9102S, RRID: AB_330744), phosphorylated proto-oncogene tyrosine-protein kinase Src (Tyr416) (1:1000, CST Cat# 2101S, RRID: AB_331697), and total proto-oncogene tyrosine-protein kinase Src (Tyr416) (1:1000, CST Cat# 2109S, RRID: AB_2106059). Blots were incubated overnight with primary antibodies and then with horseradish peroxidase-linked secondary antibody for 1 hour, including a goat anti-rabbit IgG (1:2000, CST #7074, RRID: AB_2099233), a goat anti-rat IgG (1:4000, ThermoFisher Cat# 31470, RRID: AB_228356), and a chicken anti-goat (1:2000, R&D Cat# HAF019, RRID: AB_573132).

### Immunocytochemistry

Cells were grown in 18-well chambered coverslips (Ibidi Cat# 81816) until 80% confluence, then washed and fixed in cold 4% paraformaldehyde in PBS for 15 minutes at room temperature. Cells were washed in 0.05% PBS-Tween 20 and permeabilized in 0.3% PBS-Tween 20 for 3 minutes. After 2 additional washes, the cells were blocked for 1 hour in 10% donkey serum and 1% BSA in wash buffer. The primary antibodies used were a rabbit GHR antibody (10 ug/mL, Thermo Fisher Scientific Cat# MA5-49857, RRID: AB_3092625) and a rabbit isotype control antibody (R&D Systems Cat# AB-105-C, RRID: AB_354266). Primary antibodies were diluted in 1% BSA in wash buffer and added to the cell chamber for an overnight incubation. The next day, the chambers were washed twice, and the fluorescent secondary antibody Alexa Fluro 555 (Thermo Fisher Scientific Cat# A32732, RRID: AB_2633281) was diluted to 5 µg/mL in 1% BSA and wash buffer, then incubated for 1 hour. After further washes, F-actin was stained by incubating for 40 minutes with a phalloidin labeling probe conjugated to Alexa Fluor 488 (1:1000, ThermoFisher Cat# A12379) in PBS. The nuclei were stained by a 2-minute incubation with DAPI (1:1000, FluroPure, ThermoFisher Cat# D211490) in PBS. Finally, a few drops of nonhardening mounting medium (Ibidi Cat# 50001) were added to each chamber to cover the cells, and the coverslips were stored at 4 °C until imaging.

### Statistical Analysis

The normality and variance of each dataset were first assessed using the Shapiro-Wilk test and either the F-test or the Brown-Forsythe test. These outcomes determined the choice of statistical method. Normally distributed data with equal variances were analyzed with an unpaired 2-tailed *t*-test, while data with unequal variances were analyzed with Welch's *t*-test. For nonparametric data with equal variances, the Mann–Whitney U test was applied; for nonparametric data with unequal variances, the Kolmogorov–Smirnov test was used.

For comparisons among more than 2 groups, normally distributed data with equal variances were analyzed using a standard 1-way ANOVA with the Tukey-Kramer post hoc test, and results were displayed using a capital letter display. Nonparametric data, regardless of variance homogeneity, were analyzed using the Kruskal–Wallis test with Dunn's post hoc analysis, and results were presented in a lower-case compact letter display. The change in the normalized LVD percentage over time after the surgery was assessed between the groups using a mixed-effects 2-way ANOVA with multiple comparisons correction using the Tukey method to assess the impact of time, genotype, and their interaction on LVD. Similarly, a mixed-effects 2-way ANOVA was used to assess the effect of time, genotype, and their interaction on the pumping rate of the contralateral collecting vessel at the beginning and end of the experiment. If the ANOVAs did not show significant variation among the groups, then the postanalysis was not performed. If the primary analysis indicated statistical significance, the post hoc *P*-values were displayed (26). *P*-values from each test were displayed on the corresponding graphs, colored red when statistically significant (*P* < .05) and black when nonsignificant (*P* ≥ .05).

## Results

### The Lymphatic Pumping Rate Is Positively Correlated to Chronic GH Action, But Either Direction of Transgenic GH Perturbation Delays Wound Healing

**Figure 2.**
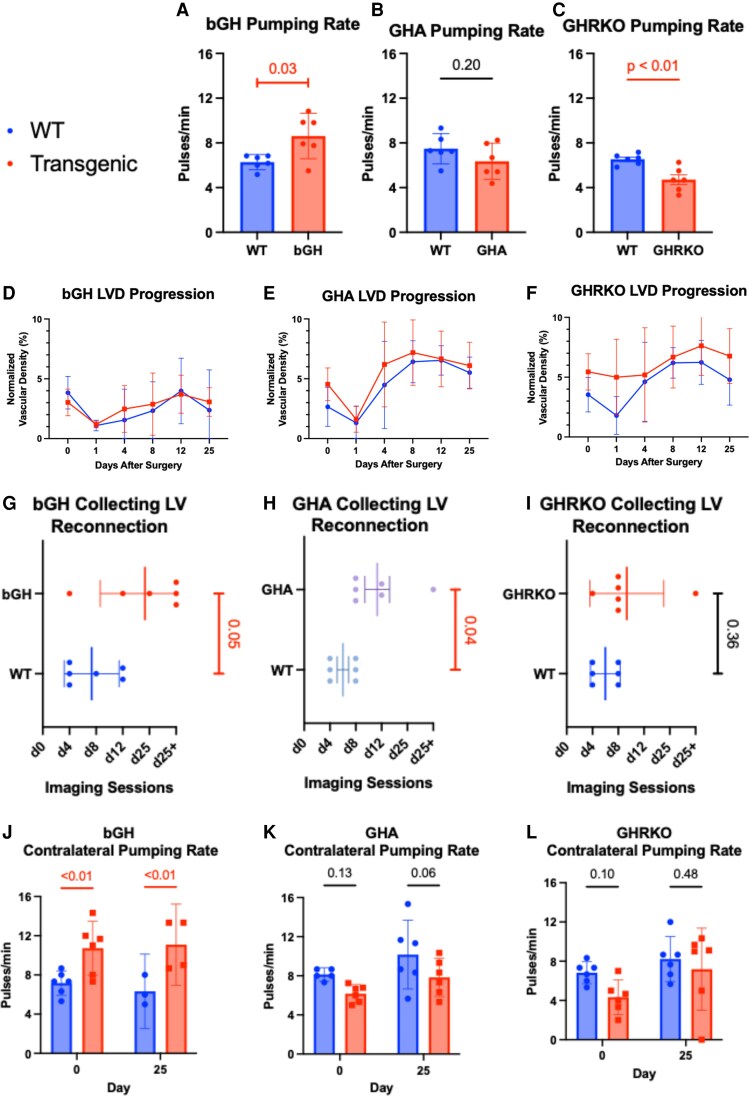
Quantitative analysis of lymphatic vessel parameters using NIRF imaging in mice with perturbed GH signaling at baseline and during wound healing. The lymphatic pumping rates of the bGH, GHA, and GHRKO mice were compared to their WT littermates using the Welsch's *t*-test (A) and unpaired *t*-test (B, C). Repeated measurements of lymphatic vascular density for 1 month after unilateral conducting lymphatic vessel transection (D, E, F). The difference between the days when the conducting vessel reconnected, as indicated by continuous flow from the inguinal to the axillary lymph node through the 1 vessel, was assessed using the Mann–Whitney U-test (G, H, I). The effect of genotype and the surgery on the pumping rate of the untransected vessel on the opposite (contralateral) flank of the mouse was assessed with a mixed-effects 2-way ANOVA with multiple comparisons testing using Fisher's least significant difference test (J, K, L). The *P*-value represents the statistical significance of the difference between the transgenic animals and their WT littermates. The bar graphs represent mean fold change ± 95% CI. A red line and a number signify a statistical change (*P*-value ≤ .05) (n = 6). Abbreviations: bGH, bovine GH; CI, confidence interval; GHA, GH antagonist; GHRKO, GH receptor knockout; NIRF, near-infrared fluorescent; WT, wild-type.

In Fig. 2, we calculated each animal's pumping rate by taking the average of the measurements for the left and right collecting lymphatic vessels. The pumping rate was positively correlated to GH action as bGH mice had the fastest pumping rate at 8.6 ± 0.7 pulses/min, followed by the averaged wild-type pumping rate at 6.8 ± 1.0 pulses/min, then the GHA at 6.4 ± 1.5 pulses/min, and finally the GHRKO mice at 4.7 ± 1.1 pulses/min. When compared to their wild-type littermates, the bGH mice pumped significantly faster (*P* = .03), and the GHRKO mice pumped significantly slower (*P* < .01). The GHA mouse's pumping rate was slower but not significantly so (*P* = .20).

Transecting the conducting lymphatic vessel rerouted ICG-laden lymph through alternative vessels until lymphangiogenesis reestablished flow through the conducting lymphatic vessel. The day the conducting vessel lumen was reconnected was identified through sequential NIRF imaging sessions to determine when flow from the inguinal to axillary lymph node was restored. We found that disrupted GH action nearly doubled the time required for reconnection in bGH (*P* = .05) and GHA (*P* = .04) mice. In addition to reconnection time, we quantified lymphangiogenesis by using NIRF imaging to measure the flank LVD. Although we found that all mice exhibited similar lymphangiogenic responses, we observed that the LVD before wounding was higher in the GHA and GHRKO animals and lower in the bGH.

### Chronic/Elevated GH Action Is Negatively Correlated with the Expression of Lymphatic-specific Proteins in Multiple Tissues

**Figure 3.**
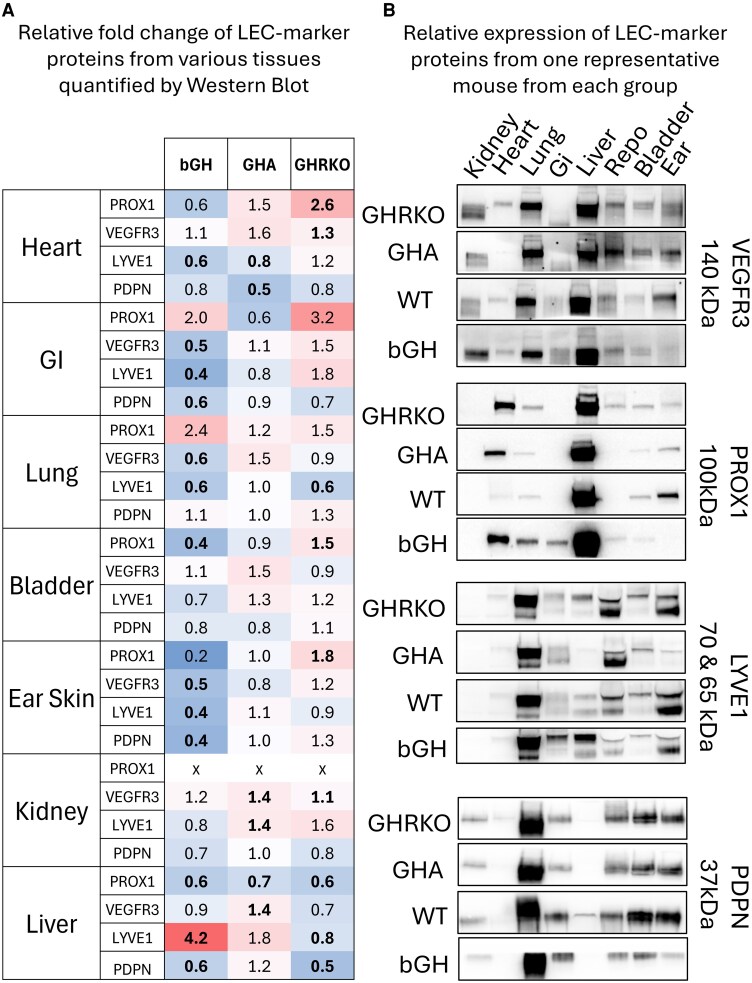
Chronic GH action is negatively correlated with protein markers of LEC in multiple tissues in Western blot analysis. (A) Western blots were performed on various tissues for LEC markers in all 3 genotypes and their respective littermates. Target protein expression was normalized to the total protein staining in the lane and represented as a fold change. The cell color was determined by the fold change. Unpaired *t*-testing was used to assess whether there was a statistically significant difference between the transgenic mouse strains and their WT littermates. If there was a statistically significant difference between the 2 groups (*P*-value ≤ .05), the fold change was bolded (heart n = 3, other organs n = 5-6). The gastrointestinal sample was collected from the jejunum. (B) To assess variation in the expression of LEC marker proteins across organs, a single Western blot was performed on organs taken from 1 individual mouse in each group (GHRKO, GHA, WT GHA littermate, and the bGH) (n = 1). Abbreviations: bGH, bovine GH; GHA, GH antagonist; GHRKO, GH receptor knockout; LEC, lymphatic endothelial cells; WT, wild-type.

To assess how chronic GH perturbation alters the expression of LEC marker proteins, we performed Western blots on multiple organs collected from the transgenic mice used in the wound-healing study. As seen in Fig. 3, nearly every organ of the bGH animals exhibited decreased LEC-protein expression. This was most evident in the ear and small intestine, where all 3 membrane markers of LECs were significantly reduced; the trend was also observed in the heart, lung, and bladder, albeit not as robustly. However, when examining the blots from GHA and GHRKO animals, no ubiquitous trend was observed, and fewer significant changes were found. In the heart, there was a significant decrease in expression, whereas in the small intestine and the ear, there appeared to be an increase in LEC markers with decreased GH action. Two organs that opposed this trend were the liver and the kidney. In the liver, the GHRKO animals exhibited significantly decreased LEC marker expression, whereas the bGH had increased expression. In the kidney, both increased and decreased GH action was associated with increased expression of the LEC markers.

We chose a random mouse from each genotype to measure expression of lymphatic marker proteins across multiple organs on a single Western blot, as shown in Fig. 3. The relative expression of podoplanin and LYVE1 was highest in the lung, followed by the uterus, bladder, and ear skin, a trend observed across genotypes. Similarly, the liver and lungs expressed the highest levels of vascular endothelial growth factor receptor 3 across genotypes. In wild-type mice, the expression of prospero homeodomain protein 1 was the highest in the liver, followed by the ear and skin. However, the second-highest expression in the bGH, GHA, and GHRKO mice was in the heart.

**Figure 4.**
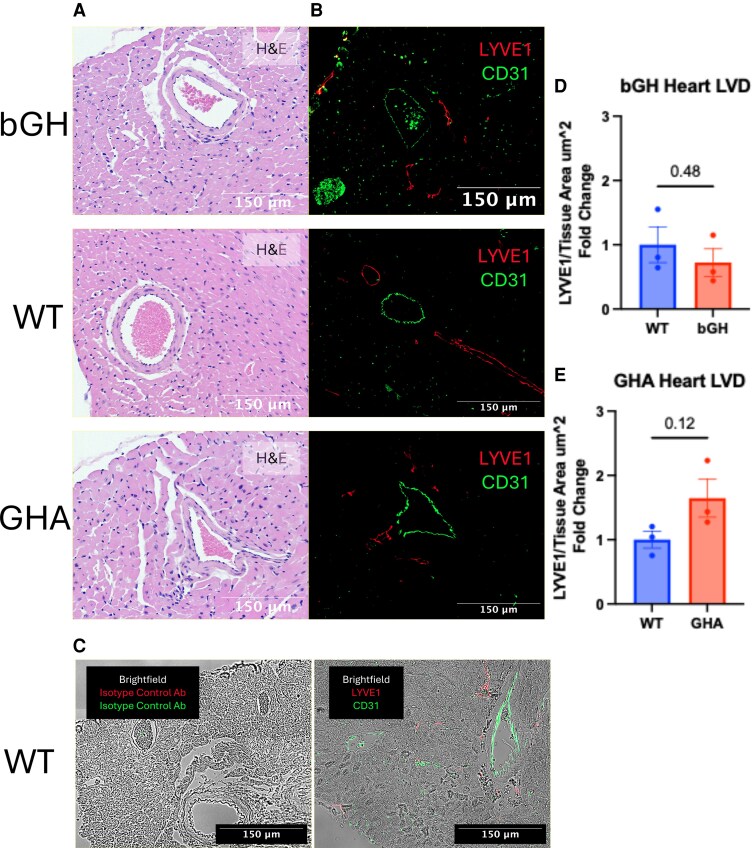
LVD quantification using IHC on the hearts of transgenic mice with perturbed GH signaling. (A) Hematoxylin and eosin staining of mouse heart sections from each genotype. (B) Representative images from IHC staining using 2 primary antibodies against LYVE1 and CD31. Fluorescent detection using a red Cy5 or green Alexa488 fluorophore bound to a secondary antibody was used to visualize LYVE1 and CD31, respectively. (C) Fluorescent IHC combined with brightfield imaging using the previously mentioned antibodies toward specific protein targets (LYVE1 and CD31) and using isotype control antibodies. (D, E) The LYVE1-positive area and total area were measured using a pixel classifier in QuPath across 3 to 5 sections per mouse from 3 mice per group (n = 3). The mean LVD ± SEM is shown in each bar graph. Group comparisons were performed using an unpaired *t*-test, with *P*-values displayed above the graph. Abbreviations: IHC, immunohistochemistry; LVD, lymphatic vascular density; LYVE1, lymphatic vessel-hyaluronic acid receptor 1.

**Figure 5.**
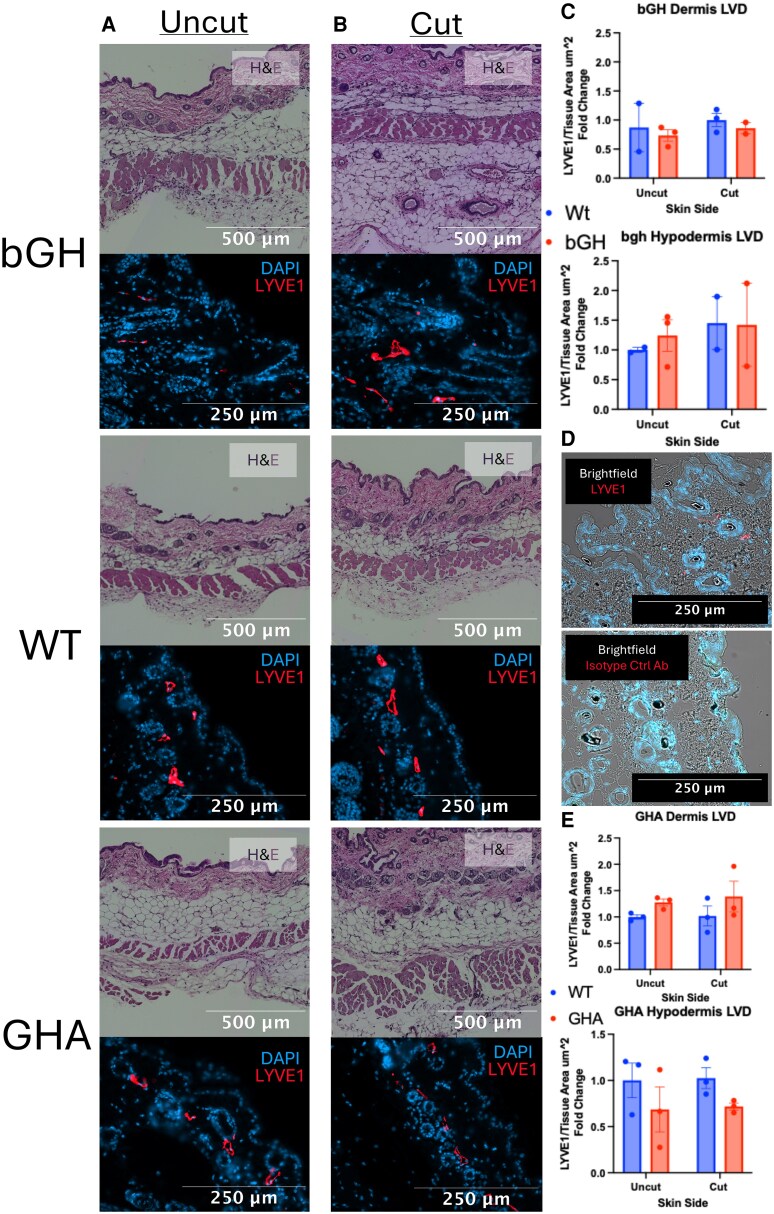
Skin histology of the uncut and cut sides 1 month after surgery in transgenic mice with altered GH signaling. Representative H&E and fluorescent IHC images of cut (A) and uncut (B) skin from the bGH, WT littermate from the GHA cohort, and GHA mice. The H&E image is of the full skin thickness at 10×, while the IHC image is of the dermis at 20×. The IHC staining used 1 primary antibody against LYVE1 and 1 secondary conjugated to the Cy5 fluorophore along with DAPI staining of the nuclei. The LYVE1-positive area and total area were measured using a pixel classifier in QuPath across 3 to 5 sections per mouse from 2 to 3 mice per group (n = 2-3). To assess statistical differences between the cut and uncut sides and between WT and transgenic animals, a mixed-effects 2-way ANOVA was used. No statistical difference in LVD was observed between WT and transgenic animals in the dermis or hypodermis or between the cut and uncut sides in the bGH (C) and GHA (E) groups; therefore, *P*-values from post hoc analysis were not calculated or displayed. Bar graphs represent mean ± SEM. (D) Brightfield imaging was combined with fluorescent imaging of LYVE1 staining or isotype control antibody staining to assess for antibody specificity. Abbreviations: bGH, bovine GH; DAPI, 4',6-diamidino-2-phenylindole; GHA, GH antagonist; H&E, hematoxylin and eosin; IHC, immunohistochemistry; LVD, lymphatic vascular density; LYVE1, lymphatic vessel-hyaluronic acid receptor 1; WT, wild-type.

To confirm the negative trend between GH action and LVD found with NIRF imaging, we performed fluorescent immunohistochemistry on the skin from both sides of the animal and the heart for LYVE1, as seen in Figs. 4 and 5. When divided by the total area measured, this served as another measure of LVD. Although none of the measures reached statistical significance, we found that increased GH action negatively correlated with LVD in the heart and dermis of the skin and that decreased GH action did the opposite. Surprisingly, we found that the hypodermis of the skin displayed the opposing trend and that GH action was positively correlated with hypodermis LYVE1 staining.

### GHR Antagonism Disrupts GH-induced LEC Activation

**Figure 6.**
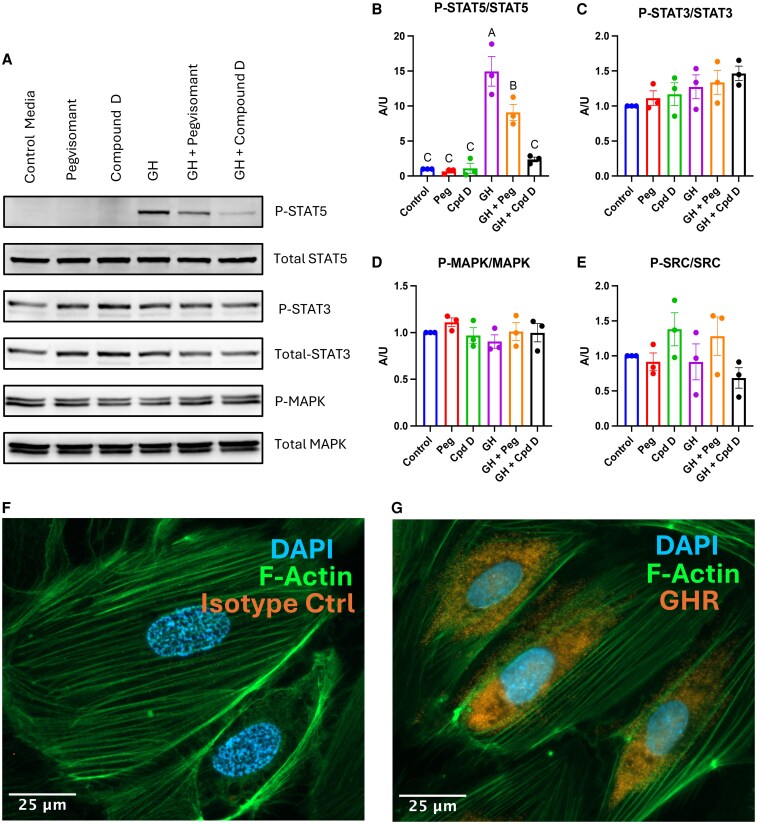
GHR activation on human LECs can be blocked with GHR antagonists. (A) Western blot analysis for intracellular signaling mediators was performed on proteins extracted from LECs treated with different combinations of GH and GHA for 10 minutes. The normalized signal of the target protein was calculated by dividing the phosphorylated signal by the total protein signal (B, C, D, E). Each treatment experiment was repeated 3 times. Bar graphs represent the mean of the 3 experiments ± SEM. The difference between the groups was assessed with an ordinary 1-way ANOVA, and post hoc analysis was performed with Tukey's multiple comparisons test. If the primary ANOVA was significant, the pairwise comparisons were displayed using the compact letter display (n = 3). Immunocytochemistry was performed on primary human dermal lymphatic endothelial cell lines for DAPI, F-actin staining using phalloidin toxin conjugated to an Alexa 488 fluorophore, and the GHR (G) or an equal concentration of isotype control antibody (F) using a 40× objective. Abbreviations: DAPI, 4',6-diamidino-2-phenylindole; GHA, GH antagonist; GHR, GH receptor; LEC, lymphatic endothelial cells.

To assess the feasibility of targeting the GHR in human LEC, we evaluated the ability of 2 GHR antagonists, pegvisomant and compound D, to block GH-induced signaling (Fig. 6). We assessed the relative phosphorylation states of 4 signaling molecules relevant to GH and LEC physiology: STAT5, STAT3, mitogen-activated protein kinase, and tyrosine-protein kinase Src. The only change observed was an increase in phosphorylated STAT5, which was markedly elevated with GH treatment. This action was significantly reduced by pegvisomant and almost entirely blocked by compound D.

**Figure 7.**
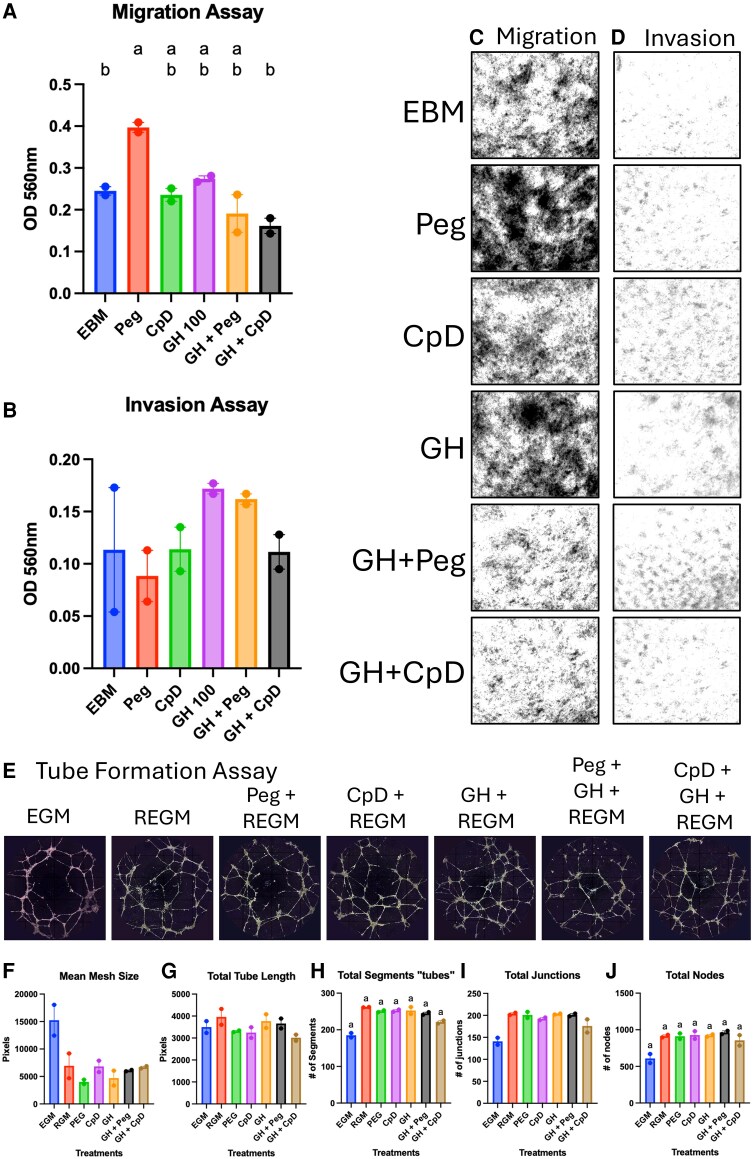
Targeting the GH receptor alters LEC physiology. Transwell assays were used to assess the impact of GH and GHA treatments on LEC migration and invasion. Cells were pretreated for 48 hours and then plated onto the transwell membrane. After incubation (18 hours for the migration assay and 36 hours for the invasion assay), the membranes were removed, dried, and imaged at 10×. Cells were then detached and quantified using a colorimetric reaction measured by spectrophotometry. Each assay was performed twice with 2 technical replicates. The OD is displayed as a bar graph representing the mean ± SEM for the migration (A) and invasion (B) assays (n = 2). Representative images of cells attached to the underside of the transwell membrane before cell quantification for the migration (C) and invasion (D) assays. To choose representative images, 5 images of each membrane were taken. The surface area covered by cells was quantified using the same Weka segmentation algorithm, and the image closest to the average is displayed. (E) Tube formation assays were performed, and images were taken at 17 hours after the cells were plated with treatment media. Images of the entire well were taken 10× using phase-contrast microscopy, and the individual images were stitched together. The effect of the treatments on various tube formation parameters measured with the Angiogenesis Analyzer plug-in for ImageJ (F, G, H, I, J). Each experiment had 2 technical replicates and was repeated twice. The bar graphs represent the mean ± SEM. The difference between the groups was assessed with the Welch 1-way ANOVA and post hoc analysis with Dunnett's T3 multiple comparisons test. Post hoc analysis and compact letter display were only generated if a significant difference was found in the ANOVA. The cells were treated in endothelial growth media, reduced endothelial growth media, pegvisomant, GH, compound D, and their combinations. Abbreviations: GHA, GH antagonist; LEC, lymphatic endothelial cells.

To assess whether GH antagonism could prevent GH-induced lymphangiogenesis, we performed migration, invasion, and tube formation assays using pegvisomant and compound D (Fig. 7). A trend emerged in which GH appeared to activate LECs to migrate or invade, and the antagonists blunted this effect. Interestingly, pegvisomant alone significantly increased migration. Although the tube formation assay yielded no statistically significant results, it appeared that the pegvisomant and compound D reduced GH action on tube formation.

## Discussion

Our findings demonstrate that chronic GH activity alters lymphatic pumping in vivo. Transgenic mice with elevated GH signaling exhibited faster lymphatic pumping rates, whereas mice with reduced or absent GH activity showed proportionally slower rates. Since vessel filling is a primary driver of lymphatic contraction frequency (27), our data suggest that chronic GH action also alters lymph flow through the lymphatic system. Given that GH increases tissue blood flow (5), our results collectively suggest that GH augments fluid movement into and out of tissues. An avenue for future work would be to compare the acute effects of GH on the lymphatic system with those of its metabolic counterpart, insulin.

We propose that the delayed wound healing observed in transgenic mice reflects the homeostatic interplay between GH and lymphatic vessels, analogous to blood vessels, in which both GH excess and deficiency drive organ dysfunction. Although chronic GH activity correlated with lymphatic pumping kinetics, both wild-type and transgenic mice showed similar trends in LVD progression after wounding. All mice had an immediate drop in LVD after vessel ligation, followed by an increase over the next 8 to 12 days and, by day 25, a decrease in LVD toward the presurgical level. This progression suggests that the surgical model induces lymphangiogenesis and regression.

To quantify tissue lymphatics, we used evidence from NIRF imaging of the skin; immunohistochemistry of the skin and heart; and Western blotting of the skin, heart, and multiple other organs. In the dermis and heart, the trend in all 3 analyses was that a chronic increase in GH action lowers the LVD, while a chronic decrease in GH action raises the LVD. The Western blots of the other organs suggest the gastrointestinal and bladder likely share this trend. In the hypodermis, this trend was reversed. The bGH appeared to have increased hypodermal LYVE1 staining, while the GHA mice had less. The hypodermis contains more lymphatic collecting vessels and fewer lymphatic capillaries than the dermis (28). Therefore, GH action could affect lymphatic capillaries differently than collecting vessels.

The transgenic mice used in this study also serve as models in aging research. In humans, a decreased lymphatic pumping rate and increased vessel permeability are now accepted as central to the aging process (29). The GHRKO mouse, created in our laboratory, holds the record for the world's longest-lived laboratory mouse (30). This mouse line, along with the GHA and bGH mice, has shown that a chronic increase in GH action is negatively correlated with lifespan, while a decrease in GH action is positively correlated with lifespan (31). The mice used in this study were 6 months old at the start. Although this is an early time point to examine age-related pathology in wild-type mice, it was nearly the halfway point for the bGH mouse's lifespan (32) and only about one-eighth of the GHRKO's lifespan (33). By considering bGH mice as the model for accelerated aging and the GHA and GHRKO mice as the models for slowed aging, our results for LVD and the quantification of lymphatic-specific proteins align with the literature: advanced age decreases LVD and the expression of lymphatic-specific proteins. These findings provide another mechanism by which transgenic mice with perturbed GH signaling model human age-related changes.

However, this was not true for our lymphatic pumping rate results. The literature shows that aging decreases the lymphatic pumping rate (34); however, we found that the long-lived GHRKO mice have slower lymphatic pumping rates than their wild-type counterparts. One possible cause for this discrepancy is that the GHA and GHRKO mice are obese, and the bGH mice are lean. Obesity has been shown to reduce lymphatic pumping but also cause LVD rarefaction in mice (35). Our results suggest that the decreased lymphatic pumping rate associated with obesity might not be pathological, as the GHA and GHRKO mice, which serve as models of healthy aging, are obese and have decreased lymphatic pumping rates compared to their wild-type counterparts. It is worth noting that this study only involved female mice. Since primary lymphatic disorders more commonly affect females than males (36), we initially focused on females and now plan to repeat these measurements in male mice.

Lastly, we demonstrated that the GHR on human LECs is pharmacologically targetable using both Food and Drug Administration-approved and novel GHR antagonists, resulting in marked suppression of GH-activated STAT5 signaling. Consistent with this effect, we observed insignificant trends toward reduced GH-induced LEC migration, invasion, and tube formation. These findings suggest that GH receptor antagonism can directly modulate GH-dependent lymphatic endothelial behavior and highlight a potential novel therapeutic strategy for lymphatic dysfunction. Rather than focusing solely on suppressing proinflammatory mediators, GHR antagonism may improve chronic inflammatory states by enhancing lymphatic drainage and disrupting the self-amplifying feedback loop between impaired lymphatic function and chronic inflammation. Future research will be necessary to determine the role of GHR antagonism in specific chronic inflammatory conditions and primary lymphatic disorders.

## Data Availability

Original data generated and analyzed during this study are included in this published article or in the data repositories listed in References.
